# Phytochemical constituents and biological activities of *Salvia macrosiphon* Boiss.

**DOI:** 10.1186/s13065-020-00728-9

**Published:** 2021-01-19

**Authors:** Majid Balaei-Kahnamoei, Mahdieh Eftekhari, Mohammad Reza Shams Ardekani, Tahmineh Akbarzadeh, Mina Saeedi, Hossein Jamalifar, Maliheh Safavi, Sohrab Sam, Naghmeh Zhalehjoo, Mahnaz Khanavi

**Affiliations:** 1 Department of Pharmacognosy, Faculty of Pharmacy, Tehran University of Medical Sciences, Tehran, Iran; 2 Department of Pharmacognosy and Pharmaceutical Biotechnology,School of Pharmacy, Kermanshah University of Medical Sciences, Kermanshah, Iran; 3 Department of Medicinal Chemistry, Faculty of Pharmacy, Tehran University of Medical Sciences, Tehran, Iran; 4 Medicinal Plants Research Center, Faculty of Pharmacy, Tehran University of Medical Sciences, Tehran, Iran; 5 Persian Medicine and Pharmacy Research Center, Tehran University of Medical Sciences, Tehran, Iran; 6 Quality Control of Pharmaceuticals and Supplements Group, Pharmaceutical Quality Assurance Research Center, The Institute of Pharmaceutical Sciences (TIPS), Tehran University of Medical Sciences, Tehran, Iran; 7 Department of Biotechnology, Iranian Research Organization for Science and Technology, P. O. Box 3353-5111, Tehran, Iran; 8 Department of Biochemistry, Genetics, Nutrition and Medicine, Alborz University of Medical Sciences, Karaj, Iran; 9 Faculty of Land and Food Systems, University of British Columbia, Vancouver, BC Canada

**Keywords:** Antibacterial, Cytotoxic, Lamiaceae, *Salvia macrosiphon Boiss.*

## Abstract

*Salvia macrosiphon* Boiss. is an aromatic perennial herb belonging to the family Lamiaceae. Phytochemical studies and biological activities of this plant have been rarely documented in the literature. The current study aimed to investigate antibacterial and cytotoxic activity of different fractions of aerial parts of *S. macrosiphon*. Also, we tried to isolate and identify cytotoxic compounds from the plant. In this respect, the hydroalcoholic extract of the corresponding parts of the plant was fractionated into four fractions. Then, antibacterial and cytotoxic activity of each fraction were examined. It was found that the chloroform fraction had a good antibacterial activity against gram-positive and gram-negative bacteria. The most potent cytotoxicity was also obtained by the *n*-hexane fraction comparing with etoposide as the reference drug which was selected for the study and characterization of secondary metabolites. Accordingly, 13-epi manoyl oxide (**1**), 6α-hydroxy-13-epimanoyl oxide (**2**), 5-hydroxy-7,4'-dimethoxyflavone (**3**), and β-sitosterol (**4**) were isolated and evaluated for their cytotoxic activity. Among them, compound **1** revealed significant cytotoxicity against A549, MCF-7, and MDA-MB-231. It merits mentioning that it showed high selectivity index ratio regarding the low cytotoxic effects on Human Dermal Fibroblast which can be considered as a promising anticancer candidate.

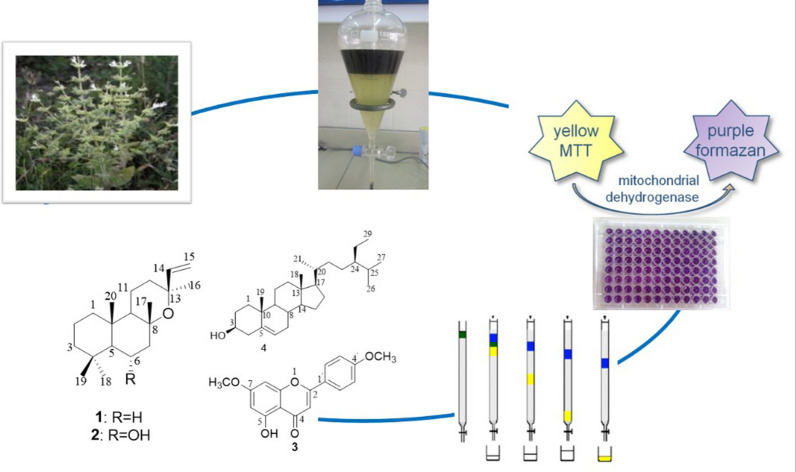

## Introduction

*Salvia* is the largest genus among the Lamiaceae family members and possesses more than 1000 species which are widely distributed around the world. The Iranian flora comprises 61 *Salvia* species, 17 of which are endemic [1]. A number of *Salvia* genus with valuable biological activities are commercially important and used as a flavoring agent in foods, cosmetics, perfumery, and pharmaceutical industries [2, 3]. The name of *Salvia *comes from the Latin word “salvare” meaning “to heal”, endorsing its medical applications for thousands of years [4]. *Salvia* species have been widely used since ancient times for the treatment of different diseases such as colds, bronchitis, tuberculosis, menstrual disorders, and haemorrhage [5]. In this respect, antiproliferative effects of *Salvia* species on human tumor cell lines [6], the efficacy of *S. miltiorrhiza* for the treatment of cardiovascular and cerebrovascular diseases [7], antimicrobial and insecticidal activities of essential oil of Turkish *S. hydrangea* [8], antioxidant, immunomodulatory, antiinflammatory, antimicrobial, and insecticidal activities of *S. mirzayanii* [9], acetylcholine esterase and melanin synthesis inhibitory activities of *S. officinalis* [10], potent cytotoxicity, antioxidant, α-amylase, and α-glucosidase inhibitory activities of essential oil of *S. syriaca* [11], antibacterial activity of *salvia officinalis* against periodontopathogens [12], and antifungal activity of *Salvia desoleana* Atzei & Picci essential oil [13] have absorbed lots of attention. Furthermore, different components isolated from *Salvia* species have shown desired biological activity, e.g. antioxidant activity of abietane diterpenoids from *Salvia barrelieri* [14], antiprotozoal activity of triterpenoids from *Salvia hydrangea* [15], and cytotoxic activity of diterpenoids isolated from *Salvia hypargeia* [16].

**Fig. 1:**
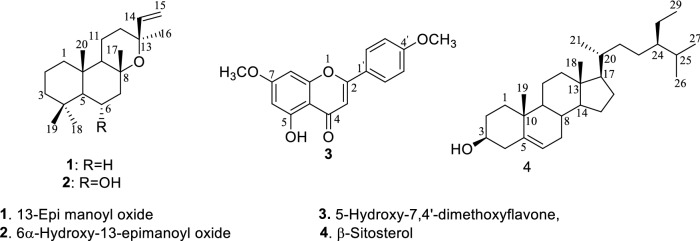
Structure of isolated compounds from *S. macrosiphon*

Herein, focusing on discovering bioactive secondary metabolites from Iranian *Salvia* species [17], we studied the aerial parts of *Salvia macrosiphon* Boiss., Wild sage known as “Marvak” in Persian [18]. The plant is an endemic species growing in the west and center of Iran, and has been used in Iranian traditional medicine as diuretic, carminative and anti-flatulent [19]. Although, *S. macrosiphon* has been commonly used in traditional medicine, a few phytochemical studies have been developed. In this respect, flavonoids and phenolic compounds (e.g. apigenin and luteolin derivatives, and rosmarinic acid), β-sitosterol, and diterpenes (e.g. 13-epi-manoyl oxide) have been isolated and reported [20, 21]. In this work, we evaluated antibacterial and cytotoxic activities of different fractions of the aerial parts of *S. macrosiphon* and focusing on the efficacy of the *n*-hexane fraction, four compounds including diterpenes (**1**, **2**), flavonoid (**3**), and steroid (**4**) were isolated and identified which one of them, compound **2** was reported for the first time for this plant (Fig. 1).

## Materials and methods

### General experimental procedures

NMR (nuclear magnetic resonance, as ^13^C-NMR, ^1^H-NMR) spectra were recorded on an Avance III spectrometer (Bruker) operating at 400.20 MHz for ^1^H and 100.63 MHz for ^13^C. Solvents for the extraction and column chromatography (CC) were of technical grade and redistilled before use. Silica gel for CC (70–230 mesh) and precoated silica gel F254 (20 × 20 cm) plates for TLC, both supplied by the Merck were used. Deuterated solvents (100 atom %D) were from Armar Chemicals. TLC plates were visualized under UV light (254 and 366 nm) and by spraying with 0.5% anisaldehyde in MeOH, followed by heating at 150 °C.

### Chemical and reagents

3-(4,5-Dimethylthiazol-2-yl)-2,5-diphenyltetrazolium bromide (MTT), Dulbecco’s Modified Eagle’s Medium (DMEM), penicillin–streptomycin, trypsin–EDTA and fetal bovine serum (FBS) were purchased from Gibco BRL (Life Technologies, Paisley, Scotland). Propidium iodide (PI), 4-6-diamidino-2-phenylindole (DAPI), dimethyl sulfoxide (DMSO), acridine orange and ethidium bromide were purchased from Sigma-Aldrich chemical Co. (St. Louis, MO, USA).

#### Plant material

The flowering aerial parts of *Salvia macrosiphon* Boiss. were collected at full flowering stage from Nurabad Mamassani, located in Fars province, Iran, in May 2014. The specimen of the plant was identified and authenticated by Professor G. Amin and deposited at the Herbarium of Faculty of Pharmacy, Tehran University of Medical Sciences (voucher specimen No.6762-TEH).

### Cytotoxic activity by MTT assay

Two different human breast cancer cell lines (MCF-7 and MDA-MB-231), lung cancer cell line (A-549) and normal cell (Human Dermal Fibroblast) were purchased from Pasture Institute of Iran, Tehran, Iran. The medium of RPMI 1640 (PAA, Germany) including sodium bicarbonate and *N*-hydroxyethylpiperazone-n-2-ethanesulfonic Acid (HEPES, Biosera, England) was used to maintain the cell lines. The medium was enriched with fetal bovine serum (FBS; Gibco, USA) and antibiotics. Then, incubated in air atmosphere enriched 5% CO_2_ at 37 °C. The cytotoxic activity of all fractions and compounds were examined by the MTT (3-[4,5-dimethylthiazole-2-yl]-2,5-diphenyltetrazolium bromide) (Sigma-Aldrich, USA) assay.

### Antibacterial activity

 In vitro antibacterial activity of all fractions was assessed against gram-positive and negative bacteria (*Staphylococcus aureus* ATCC 6538P and *Escherichia coli* ATCC 8739). Minimum inhibitory concentration (MIC) was determined by broth micro-dilution method [22]. Serial dilutions of fractions and antimicrobial agents were prepared in 96-well plates by using (Mueller-Hinton Broth) MHB, and was made in a concentration ranging from 0.125 to 64 mg/mL Mueller-Hinton Broth (MHB) medium. The standard saline solution was prepared to get inoculants turbidity solution equal to 0.5 McFarland standards. The inoculants of the microbial strains were prepared from 20 h bacterial culture that were adjusted to 0.5 McFarland standard turbidity and were further diluted (1:100) using MHB medium just before adding to the serially diluted samples. The plates were incubated for 24 h at 37 °C and MIC values were recorded as the lowest concentrations which could inhibit visible growth of microorganisms. Each experiment was done in triplicate. The ampicillin was used as the standard antibacterial agent.

### Extraction and isolation

The air-dried powdered aerial parts of *S. macrosiphon* (1.8 kg) were crushed and extracted with methanol (8 × 8 L) at room temperature for 7 days. The extract was concentrated under vacuum to afford dark green gummy residue (90.0 g). The crude methanol extract was mixed with water (700 mL) to form a suspension and partitioned successively with *n*-hexane, chloroform and ethyl acetate to yield *n*-hexane (40.0 g), chloroform (20.0 g), ethyl acetate (4.0 g) and water soluble (26.0 g) fractions.

The *n*-hexane fraction (20.0 g) was then loaded on a silica gel column (700 g, 70–230 mesh, 10 × 30 cm) and it components were separated with a gradient mixture of *n*-hexane and dichloromethane (100:0 to 0:100) as eluent, followed by increasing concentration of acetone (up to 100%) in dichloromethane. The effluents were combined to 25 fractions (F1–F25) based on TLC patterns (bands were detected on TLC under UV or by heating after spraying with 0.5% anisaldehyde in methanol).

Fraction F6 [30 mg, eluted with dichloromethane-petroleum ether (50:50)] was separated over a silica gel CC (50 g, 70–230 mesh, 1.5 × 60 cm) with a gradient mixture of chloroform/acetone (100/0 to 80/20) as eluent, to afford seven subfractions (6a-6 g). Subfraction 6f was further purified by prep. TLC [chloroform-acetone (95:5)] to afford β-sitosterol (**4**). Fraction F7 [100 mg, eluted with dichloromethane-petroleum ether (60:40)] was subjected to silica gel CC (70 g, 70–230 mesh, 2 × 80 cm) and eluted with chloroform-petroleum ether (70:30) to give apigenin-4’,7-dimethylether (**3**). From fraction F15 [3 g, eluted with dichloromethane-acetone (80:20)], crude crystals were obtained which were recrystallized from chloroform to afford 13-epi manoyl oxide (**1**). Fraction F17 [200 mg, eluted with dichloromethane-acetone (80:20)] was subjected to silica gel CC (65 g, 70–230 mesh, 2 × 75 cm) with chloroform-acetone (70:30) as eluent and subfractions (17a-17d) were obtained. Subfraction 17b was further purified by prep. TLC [chloroform-acetone (80:20)] to afford 6α-hydroxy-13-epimanoyl oxide (**2**). Their structure was elucidated by NMR spectroscopy and electrospray ionization mass spectrometry in comparison to the literature for 13-epi manoyl oxide **1** [21], 6α-hydroxy-13-epimanoyl oxide **2** [23], 5-hydroxy-7,4'-dimethoxyflavone **3 **[20], and β-Sitosterol **4** [21].

#### *13-Epi manoyl oxide* (**1**)

White amorphous powder (100 mg). mp: 96–98 °C. ^1^H NMR (CDCl_3_, 400 MHz): δ = 5.92 (1H, dd, *J* = 17.3, 10.7 Hz, H-14), 5.20 (1H, dd, *J* = 17.3, 1.1 Hz, H-15), 4.98 (1H, dd, *J* = 10.7, 1.1 Hz, H-15), 1.24 (3H, s, Me-16), 1.15 (3H, s, Me-17), 0.86 (3H, s, Me-18), 0.78 (6H, s, Me-19, Me-20). ^13^C NMR [100 MHz, CDCl_3_, based on DEPT, HMQC and HMBC experiments]: Table 1. Electron ionization mass spectrometry (EI-MS) 70 eV, m/z: 290 [M]^+^, 275, 257, 272, 257, 191, 177, 149, 137, 121, 109, 107, 95, 81, 69, 67, 57, 55, 43.

#### *6α-Hydroxy-13-epimanoyl oxide* (**2**)

White amorphous powder (5 mg). mp: 95–98 °C. ^1^H NMR (CDCl_3_, 400 MHz) δ = 5.92 (1H, dd, *J* = 7.4, 10.8 Hz, H-14), 5.19 (1H, dd, *J* = 17.4, 1.4 Hz, H-15), 5.00 (1H, dd, *J* = 10.8, 1.4 Hz, H-15), 4.41 (1H, q, *J* = 3.1 Hz, H-6), 1.34 (3H, s, Me-17), 1.24 (3H, s, Me-16), 1.18 (3H, s, Me-18), 1.17 (3H, s, Me-19), 0.96 (3H, s, Me-20). ^13^C NMR (100 MHz, CDCl_3_, based on DEPT, HMQC and HMBC experiments): Table 1. EI-MS 70 eV, m/z: 306 [M]^+^, 291, 288, 273, 150, 135, 107, 123.

#### *5-Hydroxy-7,4’-dimethoxyflavone* (**3**)

Yellow needles (5 mg). mp: 171–174 °C. ^1^H NMR (CDCl_3_, 400 MHz) δ = 12.82 (1H, s, OH-5), 7.84 (2H, d, *J* = 8.9 Hz, H-2',H-6'), 7.02 (2H, d, *J* = 8.9 Hz, H-3', H-5'), 6.58 (1H, s, H-3), 6.48 (1H, d, *J* = 2.3 Hz, H-8), 6.37 (1H, d, *J* = 2.3 Hz, H-6), 3.90 (3H, s, OMe-7), 3.88 (3H, s, OMe-4'). EI-MS m/z :298 [M]^+^, 297, 270, 269, 255, 166, 138, 132.

#### *β-Sitosterol* (**4**)

Colorless needles (7 mg). mp: 132–136 °C. ^1^H NMR (CDCl_3_, 400 MHz) δ = 5.35 (1H, d, *J* = 5.1 Hz, H-6), 3.57–3.47 (1H, m, H-3), 1.00 (3H, s, Me-19), 0.92 (3H, d, *J* = 6.4 Hz, Me-21), 0.79–0.87 (9H, m, Me-26, Me-27, Me-29 ), 0.68 (3H, s, Me-18). ^13^C NMR (100 MHz, CDCl_3_, based on DEPT, HMQC and HMBC experiments): Table 1. EI-MS m/z :414 [M]^+^, 396, 381, 329, 303, 273, 255, 231, 213.

**Table 1:** ^13^C NMR of compound **1**, **2** and **4**, based on DEPT, HMQC, HMBC experiments

Position	1	2	4
	δ _C_ (ppm)	δ _C_ (ppm)	δ _C_ (ppm)
1	39.2	41.9	37.3
2	18.9	19.1	31.7
3	42.0	44.0	71.8
4	33.2	33.7	42.3
5	56.1	57.1	140.8
6	20.4	67.2	121.7
7	44.9	51.1	31.6
8	74.7	73.3	31.9
9	61.7	61.9	50.1
10	39.6	39.4	36.5
11	18.4	18.3	21.1
12	44.9	45.4	39.8
13	73.3	73.2	42.2
14	147.6	145.1	56.8
15	110.7	110.4	24.3
16	26.2	25.8	28.3
17	24.1	23.9	56.1
18	33.2	32.4	12.0
19	21.5	22.8	19.4
20	15.4	15.8	36.1
21			18.8
22			33.9
23			26.1
24			45.8
25			29.1
26			19.0
27			19.8
28			23.1
29			11.8

## Results and discussion

### Cytotoxic activity

Cancer is the second leading cause of death and responsible for approximately 13% of mortality in the world. The current anti-cancer drugs have shown undesirable side effects, hence, developing novel, efficient, and safe dugs is definitely in high demand [24, 25]. One of the efficient approaches to new drugs is screening herbal extracts [26]. In the present study, *S. macrosiphon* was selected for the possible cytotoxic activity. For this purpose, different fractions of aerial parts of the plants including *n*-hexane, chloroform, ethyl acetate, and water-soluble fractions were screened towards lung cancer cell line (A549) and breast cancer cell lines (MCF-7 and MDA-MB-231) as well as normal cell, human dermal fibroblasts (HDF), using MTT assay comparing with etoposide as a standard drug (Table 2). The inhibitory concentration, 50% (IC_50_) values (µg /mL) were calculated by linear regression analysis, expressed in mean ± SD.

**Table 2:** In vitro cytotoxic activity of extracts of *S. macrosiphon *on cancerous cell lines (A549, MCF-7, MDA-MB-231)

Fractions	IC_50_ (µg/mL)			
	A549	MCF-7	MDA-MB-231	HDF
*n-*Hexane	20.89 ± 0.35	10.24 ± 0.15	20.98 ± 0.25	26.90 ± 1.24
Chloroform	22.87 ± 2.56	11.72 ± 1.56	25.67 ± 2.78	113.50 ± 5.24
Ethyl acetate	169.80 ± 3.56	76.43 ± 2.78	157.00 ± 6.78	189.50 ± 7.24
Methanol	344.96 ± 8.78	805.34 ± 10.45	589.00 ± 10.67	> 800
Etoposide	16.58 ± 0.78	22.08 ± 0.39	20.30 ± 0.21	92.70 ± 1.20

According to calculated IC_50_ values reported in Table 2, *n*-hexane and chloroform fractions depicted much higher cytotoxicity than ethyl acetate and water-soluble fractions. However, *n*-hexane fraction was found to be more potent than chloroform fraction in such a manner that it showed IC_50_s = 20.89, 10.24, 20.98, and 26.90 µg/mL against A549, MCF-7, MDA-MB-231, and HDF, respectively. Although the cytotoxic activity of *n*-hexane fraction towards A549 was a little lower than etoposide on the same cell line (IC_50_ = 16.58 µg/mL), its activity towards MDA-MB-231 (IC_50_ = 20.30 µg/mL) was as the same as etoposide (IC_50_ = 20.98 µg/mL). It merits mentioning that cytotoxicity of *n*-hexane fraction against MCF-7 (IC_50_ = 10.24 µg/mL) was significantly higher than etoposide (IC_50_ = 22.08 µg/mL). Apart from high cytotoxicity against MCF-7, the higher the selectivity index (SI) ratio (2.6) was calculated comparing with those obtained for A549 and MDA-MB-231. SI indicates the cytotoxic selectivity for an agent against cancer cells versus normal cell. The greater the SI value is, the more toxic the agent is against cancerous cells but safe against HF [27].

**Table 3:** Cytotoxicity of isolated compounds on cancerous (A549, MCF-7, MDA-MB-231) and normal (HDF) cell lines

Compounds	IC_50_ values (µM) and selectivity index^a^ (in parentheses)			
	A549	MCF-7	MDA-MB-231	HDF
1	19.37 ± 1.96 (17.4)	15.79 ± 0.35 (21.4)	22.24 ± 1.72 (15.2)	337.58 ± 9.20
2	170.03 ± 11.63	98.03 ± 11.27	119.51 ± 5.09	> 653
3	469.23 ± 22.75	428.05 ± 23.12	469.80 ± 23.12	> 971
4	> 100	> 100	> 100	nd
Etoposide	28.17 ± 1.32 (5.6)	37.51 ± 0.66 (4.2)	34.49 ± 0.36 (4.6)	157.50 ± 2.04
^a^The selectivity index was determined as IC_50_ value for human normal fibroblast (HDF)/IC_50_ for cancerous cell line. *nd* not detected				

With these results in hand, the *n*-hexane fraction was candidate for further phytochemical analysis leading to isolation and identification of compounds **1**–**4**. It worth mentioning that there is no report for the cytotoxicity of those compounds except β-Sitosterol which depicted no activity [28]. Cytotoxic evaluation of isolated compounds (Table 3) demonstrated much higher cytotoxicity of 13-epi manoyl oxide (**1**) against all cancerous cell lines. Compound **1** showed activity against A549, MCF-7, and MDA-MB-231 with IC_50_s = 19.37, 15.79, 22.24 µM comparing with etoposide with IC_50_s = 28.17, 37.51, and 34.49 µM, respectively. It should be mention that promising safety was obtained in the case of compound **1** and SI ratio was calculated as 17.4, 21.4, and 15.2 on A549, MCF-7, and MDA-MB-231 comparing with those calculated for etoposide with SI ratio of 5.6, 4.2, and 4.6. (Table 3) indicating its high efficacy with negligible side effects on normal cells.

As can be seen in Table 3, it can be concluded that 13-epi manoyl oxide **1** not only showed less toxicity towards HDF than MCF-7, but also showed more selectivity than etoposide confirming that it could be considered as a potent  candidate in anticancer drugs research and development.

### Antibacterial activity

The antibacterial activity of *n*-hexane, chloroform, and ethyl acetate fractions of aerial parts of *S. macrosiphon* was evaluated against gram-positive bacterium (*S. aureus*) and gram-negative bacterium (*E. coli*) based on the agar microdilution method (Table 4). It was found that all fractions depicted moderate to good antibacterial activity with minimum inhibitory concentration (MIC) values ranged from 0.61 to 2.5 mg/mL comparing with ampicillin with MIC values of 0.5 and 0.12 µg/mL against *S. aureus* and *E. coli*, respectively. Among fractions, the chloroform fraction exhibited more potent activity against both strains (MIC = 0.61 mg/mL) and ethyl acetate fraction showed lower activity than chloroform fraction against both strains (MIC = 0.80 mg/mL). However, the *n*-hexane fraction with MIC values of 1.25 and 2.50 mg/mL against *S. aureus* and *E. coli*, respectively was the weakest antibacterial fraction.

Antibacterial activity of *S. macrosiphon* has not been fully investigated, however, Javidnia et al. reported the activity of methanolic extract of the plant against *S. aureus* and *E. coli* [29] which showed MIC values of 1 and 0.5 mg/mL, respectively. It seemed that chloroform and ethyl acetate fractions were more potent than the methanolic extract against *S. aureus*. However, the methanolic extract was more potent than three fractions against *E. coli*.

**Table 4:** Minimum inhibitory concentration (MIC) of fractions of *S. macrosiphon* against selected bacteria

Microorganism	Fractions (MIC (mg/mL))			Ampicillin (MIC (µg/mL))
	*n*-Hexane	Chloroform	Ethyl acetate	
*S. aureus*	1.25	0.61	0.80	0.50
*E. coli*	2.50	0.61	0.80	0.12

## Conclusions

In conclusion, we investigated phytochemical analysis and biological activities of aerial parts *S. macrosiphon*. Antibacterial evaluation of *n*-hexane, chloroform, and ethyl acetate fractions of the plant against *S. aureus* and *E. coli* indicated good activity with MIC values ranging from 0.61 to 2.5 mg/mL. Further studies were devoted to the investigation of cytotoxic activity. Evaluation of all fractions against A549, MCF-7, and MDA-MB-231demonstrated very good efficacy of the *n*-hexane fraction leading to phytochemical analysis and cytotoxic evaluation of this fraction for the first time. Among four isolated compounds (13-epi manoyl oxide **1**, 6α-hydroxy-13-epimanoyl oxide **2**, 5-hydroxy-7,4'-dimethoxyflavone **3**, and β-sitosterol **4**); compound **1** was found as effective as etoposide against A549 and MDA-MB-231 and depicted higher activity than the reference drug, against MCF-7. Another good point comes back to the higher SI ratio of compound **1** for all cancerous cell lines and normal cell (HDF) comparing with etoposide verifying its efficacy and safety. It seems that the *n*-hexane fraction and also chloroform fraction of *S. macrosiphon* can be considered for comprehensive investigations to provide an herbal anticancer agent.

## Data Availability

The datasets generated during and/or analysed during the current study are available from the corresponding author on reasonable request.

## Abbreviations

IC_50_: The half maximal inhibitory concentration
^1^H NMR: Proton nuclear magnetic resonance
^13^C NMR: Carbon-13 nuclear magnetic resonance
*S. macrosiphin*: *Salvia macrosiphon*
CC: Column Chromatography
TLC: Thin-layer chromatography
DEPT: Distortionless Enhancement by Polarization Transfer
HMQC: Heteronuclear multiple quantum correla
COSY: Correlation spectroscopy
HMBC: Heteronuclear multiple bond correlation
SD: Standard deviation
SI: Selectivity index
UV: Ultraviolet radiation
MeOH: Methanol
MTT: 3-(4,5-Dimethylthiazol-2-yl)-2,5-diphenyltetrazolium bromide
DMEM: Dulbecco’s Modified Eagle’s Medium
FBS: Fetal bovine serum
PI: Propidium iodide
DAPI: 4-6-diamidino-2-phenylindole
DMSO: Dimethyl sulfoxide
MIC: Minimum inhibitory concentration
